# Global–Local Feature Fusion Network for Remote Sensing Image Change Detection in Open-Pit Mining Areas

**DOI:** 10.3390/s26103128

**Published:** 2026-05-15

**Authors:** Zhewen Zheng, Jianjun Yang, Guanghui Lv, Qiqi Li, Yuze Wang

**Affiliations:** 1 School of Ecology and Environment, Xinjiang University, Urumqi 830017, China; marshal425@126.com (Z.Z.); wyzwp78@163.com (Y.W.); 2 School of Energy and Ming Engineering, China University of Mining and Technology-Beijing, Beijing 100083, China; liqiqi_cumtb@163.com

**Keywords:** change detection, remote sensing, multi-scale features, mining area ecological monitoring, global–local feature fusion

## Abstract

Change detection in open-pit mining areas from remote sensing imagery is of great importance for mining supervision, ecological monitoring, and restoration planning. Nevertheless, mining-related changes usually exhibit multi-scale patterns, irregular boundaries, and fragmented spatial distributions, which make accurate detection difficult. Existing CNN- and Transformer-based methods often cannot effectively balance global context perception and local detail preservation, resulting in incomplete boundary extraction and insufficient sensitivity to subtle changes. To overcome these limitations, we propose GLMECD-Net, a Global–Local Multi-scale Cross-fusion Enhanced Change Detection Network for remote sensing image change detection in open-pit mining areas. Specifically, a Siamese encoder is used to extract hierarchical bi-temporal features, while a Global–Local Feature Mixing Embedding (GLME) module is introduced to jointly capture long-range contextual information and local spatial details. Furthermore, multi-scale feature aggregation and cross-temporal feature fusion are employed to improve change representation and boundary recovery. Experimental results on mining area datasets show that the proposed method achieves 71.66% Precision, 83.78% OA, 77.53% F1-score, and 53.82% IoU. The results demonstrate that GLMECD-Net provides effective and robust performance for detecting complex and subtle changes in open-pit mining areas.

## 1. Introduction

**Figure 1:**
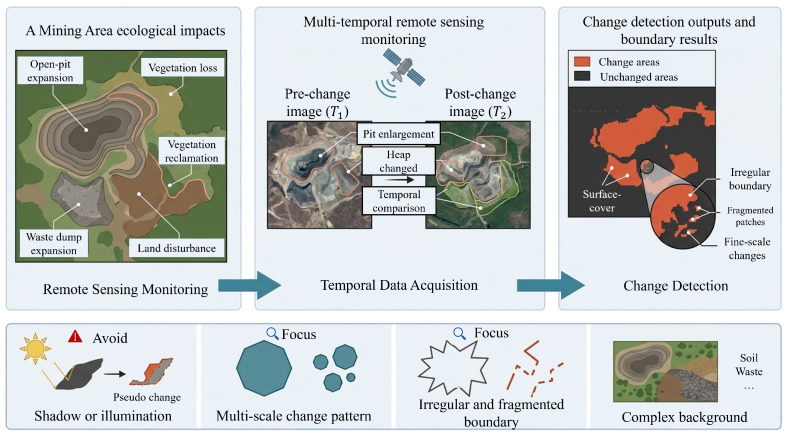
Deep learning-based change detection in mining areas enables accurate identification of semantic changes in land surface cover under complex and heterogeneous mining scenarios, providing practical support for effectively monitoring and mitigating mining-induced environmental degradation.

Open-pit mining has made significant contributions to regional economic development, but it has also caused substantial ecological and environmental damage, such as vegetation degradation, terrain disturbance, waste dump expansion, and soil erosion [1,2]. Monitoring land-use and land-cover changes in mining areas is therefore essential for ecological assessment, restoration planning, and sustainable land management [3]. Remote sensing change detection (CD), which identifies changes from bi-temporal imagery, offers an efficient and non-destructive means to support such monitoring tasks [4,5,6]. The objective of CD in mining areas is to identify surface cover transitions under complex and heterogeneous conditions as illustrated in Figure 1.

Despite recent progress, accurate CD in mining areas remains particularly challenging because the spatial organization of mining-induced changes is substantially different from that of more regular urban or agricultural scenes. Mining landscapes often contain large excavation zones, narrow transition belts, scattered reclamation parcels, and irregular waste dump boundaries that coexist within the same image [7]. Such changes are not only highly heterogeneous in scale and shape, but are also frequently embedded in spectrally confusing backgrounds. In addition, shadows, seasonal appearance differences, illumination variation, and movable objects may introduce pseudo changes that are visually similar to real land-cover transitions, thereby increasing false alarms [8]. These characteristics imply that mining area CD requires both precise local discrimination and reliable global contextual reasoning, rather than relying on a single type of feature modeling alone [9,10].

Deep learning has significantly advanced remote sensing CD. FCN-based and U-Net-based models provide strong dense prediction capability [11,12], while attention mechanisms, dilated convolutions, and ASPP improve contextual representation and multi-scale modeling [13,14,15]. Siamese CNN-based frameworks have become widely used for bi-temporal feature extraction [16,17,18]. However, conventional CNN-based methods are still constrained by limited receptive fields and progressively localized feature interactions, which makes them less effective at modeling long-range dependencies between spatially distant but semantically related mining changes. Moreover, repeated pooling and convolutional smoothing may weaken thin boundary cues and fragmented regions, resulting in omission errors and inaccurate boundary delineation. Transformer-based approaches improve global context perception, yet they often incur high computational costs and may still fail to preserve local boundary details in complex scenes. Recent hybrid models, such as ELGC-Net [19], attempt to balance convolutional locality and Transformer-style global modeling, but their capability remains limited when dealing with fragmented change regions, irregular boundary structures, and complex cross-scale interactions in mining environments.

From a mechanism perspective, the above challenges suggest three essential requirements for mining area CD. First, irregular boundaries and thin transition zones require local modeling, because the change label of boundary pixels is often determined by subtle neighborhood discontinuities and short-range structural cues. Without sufficient local detail preservation, boundary pixels are easily over-smoothed during hierarchical feature extraction. Second, fragmented and scattered change regions benefit from global contextual modeling, because isolated local patches may be ambiguous when considered independently, whereas their semantic meaning becomes clearer when their relationship to surrounding mining structures, transportation corridors, and disturbed land patterns is taken into account. Third, effective mining area CD requires stronger cross-temporal interaction than conventional feature fusion, since pseudo changes caused by illumination or seasonal variation cannot be reliably suppressed by static concatenation or simple attention weighting alone. These observations motivate a unified framework that explicitly combines local detail enhancement, global dependency modeling, and cross-temporal multi-scale fusion.

To address the above issues, we propose GLMECD-Net, a Global–Local Multi-scale Cross-fusion Enhanced Change Detection Network for remote sensing image change detection in open-pit mining areas. The proposed framework is designed according to the three requirements discussed above. Specifically, a Siamese encoder is employed to extract hierarchical features from bi-temporal images, enabling consistent representation learning across temporal domains. To improve the modeling of both large-area disturbances and fine-grained boundary variations, a Global–Local Feature Mixing Embedding (GLME) module is introduced at the encoding stage to jointly capture long-range semantic dependencies and local spatial details. Furthermore, to better handle fragmented changes and temporal inconsistency, a multi-scale cross-fusion strategy is developed to enhance feature interaction across different spatial scales and temporal phases. By combining these components within a unified architecture, GLMECD-Net aims to improve change representation, suppress pseudo changes, and achieve more accurate boundary delineation in complex mining scenes. The main contributions of this work are summarized as follows:

- A novel GLMECD-Net framework is proposed for remote sensing image change detection in open-pit mining areas, which effectively integrates global contextual modeling and local detail preservation within a unified Siamese architecture.
- A Global–Local Feature Mixing Embedding module is designed to enhance the representation of heterogeneous and fragmented change patterns by jointly capturing long-range dependencies and local spatial details.
- A multi-scale cross-fusion strategy is introduced to improve cross-temporal feature interaction and boundary delineation, leading to more discriminative change representations in complex scenes.
- We conduct extensive experiments on publicly available mining area datasets, and the results demonstrate that the proposed method achieves superior performance and robustness compared with several state-of-the-art approaches.

Experimental results demonstrate that GLMECD-Net achieves superior performance and robustness on multiple publicly available mining area change detection datasets, exhibiting clear advantages over mainstream deep learning models when dealing with highly irregular and multi-scale change patterns in mining regions. The proposed method not only provides an innovative technical route for land-use change detection in mining areas, but also offers strong technical support for ecological environment assessment and sustainable land managements [20].

The remainder of this paper is organized as follows. Section 2 reviews related algorithms for change detection. Section 3 presents the network architecture and core modules of GLMECD-Net in detail. Section 4 describes the experimental setup, results, and analysis. Section 5 provides an in-depth discussion of model performance and application prospects. Finally, Section 6 concludes the paper and outlines directions for future research.

## 2. Related Work

Remote sensing change detection methods for mining areas can be broadly grouped into traditional handcrafted-feature methods, CNN-based methods, and Transformer-based methods. However, the key difference among these paradigms is not merely the choice of backbone, but the way they model spatial context, temporal correspondence, and scale variation. In open-pit mining scenes, changes are often fragmented, irregularly bounded, and distributed across multiple spatial scales. Therefore, a practically effective change detection model should simultaneously address three tightly coupled issues: robust representation of multi-scale heterogeneous changes, effective coordination between global semantics and local structural details, and sufficient cross-temporal interaction between bi-temporal features. In the following, related studies are reviewed from this perspective, so as to clarify the architectural bottlenecks of existing methods and motivate the design of the proposed framework.

### 2.1. Traditional Change Detection Work

Traditional change detection methods can be broadly categorized into pixel-based and object-based approaches [21]. Pixel-based methods directly compute spectral or textural differences between bi-temporal remote sensing images to generate a difference map, followed by thresholding or clustering to obtain the final change map. For example, Tan et al. employed a combination of Extreme Learning Machine (ELM), Multinomial Logistic Regression (MLR), and K-Nearest Neighbors (KNN) classifiers to improve detection accuracy [22], while Celik et al. achieved unsupervised change detection by integrating Principal Component Analysis (PCA) with K-means clustering [23]. In addition, decision trees [24], change vector analysis (CVA) [25], and support vector machines (SVMs) [26] have also been widely used in conventional change detection tasks. However, these methods rely heavily on handcrafted features and are thus unable to fully capture the rich spatial and structural details present in high-resolution remote sensing imagery, leading to limited detection accuracy in complex mining area scenarios.

### 2.2. Change Detection Work Based on CNN

CNN-based methods have long been a dominant paradigm in remote sensing change detection because convolution is well suited for extracting local textures, edges, and short-range spatial patterns. Existing CNN-based change detection models can be roughly divided into three categories: encoder–decoder frameworks, Siamese feature extraction frameworks, and attention-enhanced CNN models. For example, Jiang et al. [27] introduced a collaborative attention mechanism into a Siamese encoder–decoder architecture to enhance feature interaction before decoding. Ham et al. [28] employed a deconvolutional network for change-related object extraction from UAV imagery. BiDateNet [29] adopted a U-Net-style architecture to improve dense prediction quality, while Guo et al. [30] designed a Siamese fully convolutional network for bi-temporal change analysis. Khan et al. [31] further explored weakly supervised CNN-based change detection from paired images.

In related SAR image interpretation research, CNN-based architectures have also shown strong potential for complex scene understanding. For example, the multi-scale rotation-invariant Haar-like feature integrated CNN (MSRIHL-CNN) introduces rotation-invariant handcrafted cues into a CNN framework to improve robustness to target orientation and scale variation, while the multikernel-size feature fusion CNN (MKSFF-CNN) exploits heterogeneous convolution kernels to enhance multi-scale feature representation and fusion [32,33]. Although these methods were developed for SAR target detection or recognition rather than bi-temporal mining area change detection, they provide useful insights into feature modeling under complex scattering backgrounds and multi-scale structural variations.

Overall, CNN-based methods have achieved strong performance in change detection owing to their effective local feature extraction ability. Nevertheless, their limited receptive fields make it difficult to capture long-range contextual dependencies in fragmented and heterogeneous mining scenes. Moreover, the loose coupling between global semantic modeling and local detail preservation often restricts their ability to delineate irregular boundaries and detect subtle changes. Their reliance on fixed convolutional kernels also reduces adaptability to multi-scale mining-related change patterns.

### 2.3. Change Detection Work Based on Transformers

To compensate for the limitations of CNNs in modeling long-range dependencies, researchers have introduced Transformers into the change detection domain, leveraging global attention mechanisms to enhance the joint modeling of fine-grained details and long-range contextual relationships. ChengeFormer constructs a unified architecture comprising a hierarchical Transformer encoder and an MLP decoder to efficiently capture multi-scale, long-range details [13]. Zhang et al. [34] proposed a U-shaped Swin Transformer-based network, in which a multi-stage Swin Transformer encoder and decoder framework is employed to strengthen global context exploration. Wang et al. [35] integrated a multi-scale Transformer with a convolutional block attention module (CBAM) to improve detection quality across different types of remote sensing imagery. Song et al. [36] developed a hybrid model based on a ResNet18 [37] backbone and multi-scale Swin Transformer blocks to enhance the representational power of feature maps. In the broader SAR remote sensing literature, recent models have further explored efficient feature enhancement and compensation mechanisms under complex imaging conditions. For instance, LMCNet introduces a lightweight modality compensation strategy via knowledge distillation to improve salient object detection when modality information is incomplete [38]. Although such methods are not specifically designed for open-pit mining change detection, they highlight the importance of lightweight compensation, cross-feature interaction, and robustness enhancement in complex remote sensing scenes, which are also highly relevant to mining area change analysis.

However, existing Transformer-based methods still exhibit several limitations when dealing with multi-scale, heterogeneous changes in mining areas, including insufficient representation of local fine-grained features and suboptimal fusion between global and local information, which ultimately constrains detection performance in complex mining scenarios. Beyond CNN- and Transformer-based pixel-level paradigms, recent work has explored object-level change detection to improve robustness under severe cross-temporal inconsistencies. For instance, OCD-BDA performs post-disaster building damage assessment at the object level, reducing the dependence on strict pixel-wise consistency between pre- and post-event images and demonstrating strong generalization under variations in imaging angle, resolution, and illumination [39].

In summary, existing change detection methods still face three architecture-level bottlenecks in complex mining environments. The first is insufficient adaptability to multi-scale and heterogeneous change patterns, since large-area expansion and small fragmented reclamation often coexist within the same scene. The second is inadequate coordination between global contextual modeling and local fine-grained structure preservation, which makes it difficult to accurately delineate irregular boundaries while suppressing pseudo changes. The third is imperfect cross-temporal interaction, as many existing methods do not explicitly model deep semantic correspondence between bi-temporal features across multiple scales.

## 3. Method

Given a pair of bi-temporal remote sensing images $I_{pre}$ and $I_{post}$, the proposed GLMECD-Net aims to predict a pixel-wise change map $M\in R^{H\times W}$ or a two-channel change probability map $P\in R^{H\times W\times 2}$. The network consists of a Siamese encoder, a Global–Local Feature Mixing Embedding (GLME) module embedded in the encoding stage, a multi-scale cross-fusion module, and a decoder for change map reconstruction. Specifically, the Siamese encoder first extracts hierarchical features from the bi-temporal images. Then, the GLME module enhances each stage feature by jointly modeling global contextual dependencies and local spatial details. The enhanced features are subsequently aggregated through the multi-scale cross-fusion module and finally decoded to generate the change prediction.

### 3.1. The Backbone of GLMECD-Net

**Figure 2:**
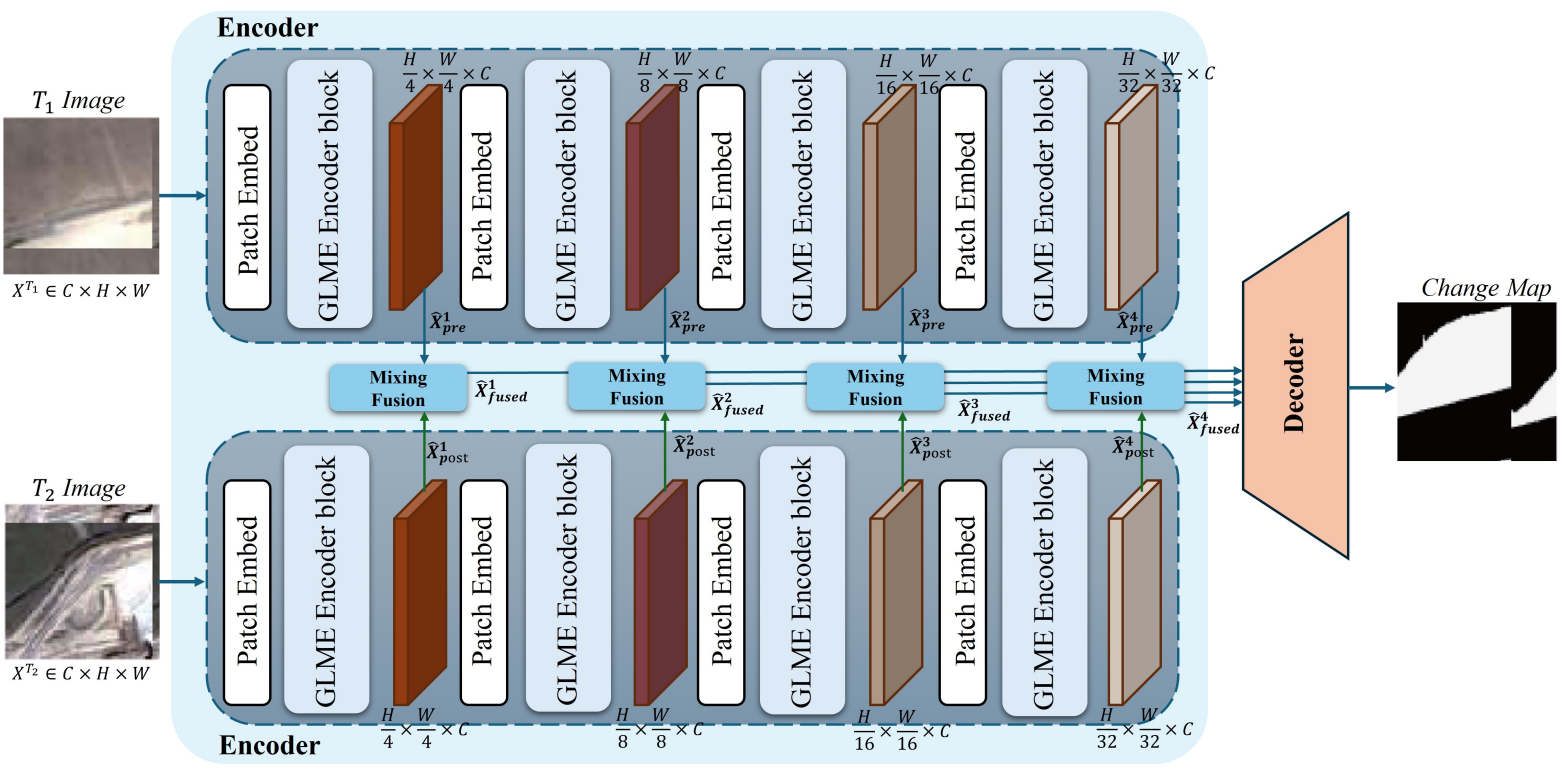
The pipeline of GLMECD-Net. The proposed network leverages the GLME modules (light blue) to effectively extract both global and local information from remote sensing images, while the Mixing Fusion module enhances cross-feature interactions between pre- and post-change representations.

As illustrated in Figure 2, GLMECD-Net adopts a Transformer-based Siamese architecture composed of an encoder, a multi-scale cross-fusion module, and a decoder. The shared Siamese encoder contains four stages, each consisting of a token-mixing block and a convolutional multilayer perceptron (MLP), and extracts hierarchical feature representations from the pre-change and post-change images. Let $F_{pre}^{i}$ and $F_{post}^{i}$ denote the feature maps generated at the *i*-th stage of the encoder. These paired features are first enhanced by the GLME module and then delivered to the multi-scale cross-fusion module for cross-temporal interaction and semantic change representation. Finally, the fused multi-scale features are decoded to reconstruct the final change map.

Let $F_{pre}^{i}$ and $F_{post}^{i}$ denote the feature maps extracted from the *i*-th stage of the shared encoder for the pre-change and post-change images, respectively, where i∈{1,2,3,4}. These features are fed into the GLME module to obtain enhanced representations ${\hat{F}}_{pre}^{i}$ and ${\hat{F}}_{post}^{i}$, which are then used for subsequent multi-scale cross-fusion.

In the encoder stage, i=1,2,3,4. The feature maps at each stage are first downsampled by a patch embedding layer and then passed through an encoder block (Figure 2) consisting of a GLME module and a convolutional MLP layer. As the core innovation of the network, the GLME module is designed to aggregate local and global contextual information, thereby improving the accuracy of change map prediction. The feature pairs produced by the encoder blocks at each stage are processed by the fusion module and subsequently forwarded to the decoder for change map generation.

The decoder adopts a multi-scale feature fusion strategy. The multi-scale fused features from the four stages $\left({\hat{F}}_{fused}^{i},i\in \left\{1,2,3,4\right\}\right)$ are concatenated along the channel dimension and fed into a 1×1 convolution layer for dimensionality reduction. A transposed convolution is then applied to increase the spatial resolution of the feature maps, followed by a residual block containing two 3×3 convolution layers to enhance feature representation. The cascade of transposed convolution and residual block is repeated twice, so that the spatial resolution of the feature maps matches that of the input images. Finally, a convolution layer outputs a two-channel prediction score map (corresponding to the unchanged and changed classes), and an argmax operation along the channel dimension is applied to obtain the binary change map.

To improve the clarity and reproducibility of the proposed architecture, the dimensions and numerical settings of the major components of GLMECD-Net are summarized in Table 1. For a pair of co-registered bi-temporal input images of size 256×256×3, the shared Siamese encoder generates four-stage feature maps with channel dimensions of 64, 96, 128, and 256, respectively. Correspondingly, the spatial resolutions of the four stages are 64×64, 32×32, 16×16, and 8×8. The same stage-wise configuration is used for both temporal branches. The enhanced and fused features are then decoded to produce a two-channel prediction map with the same spatial resolution as the input image.

### 3.2. Global–Local Feature Mixing Embedding (GLME) Module

**Figure 3:**
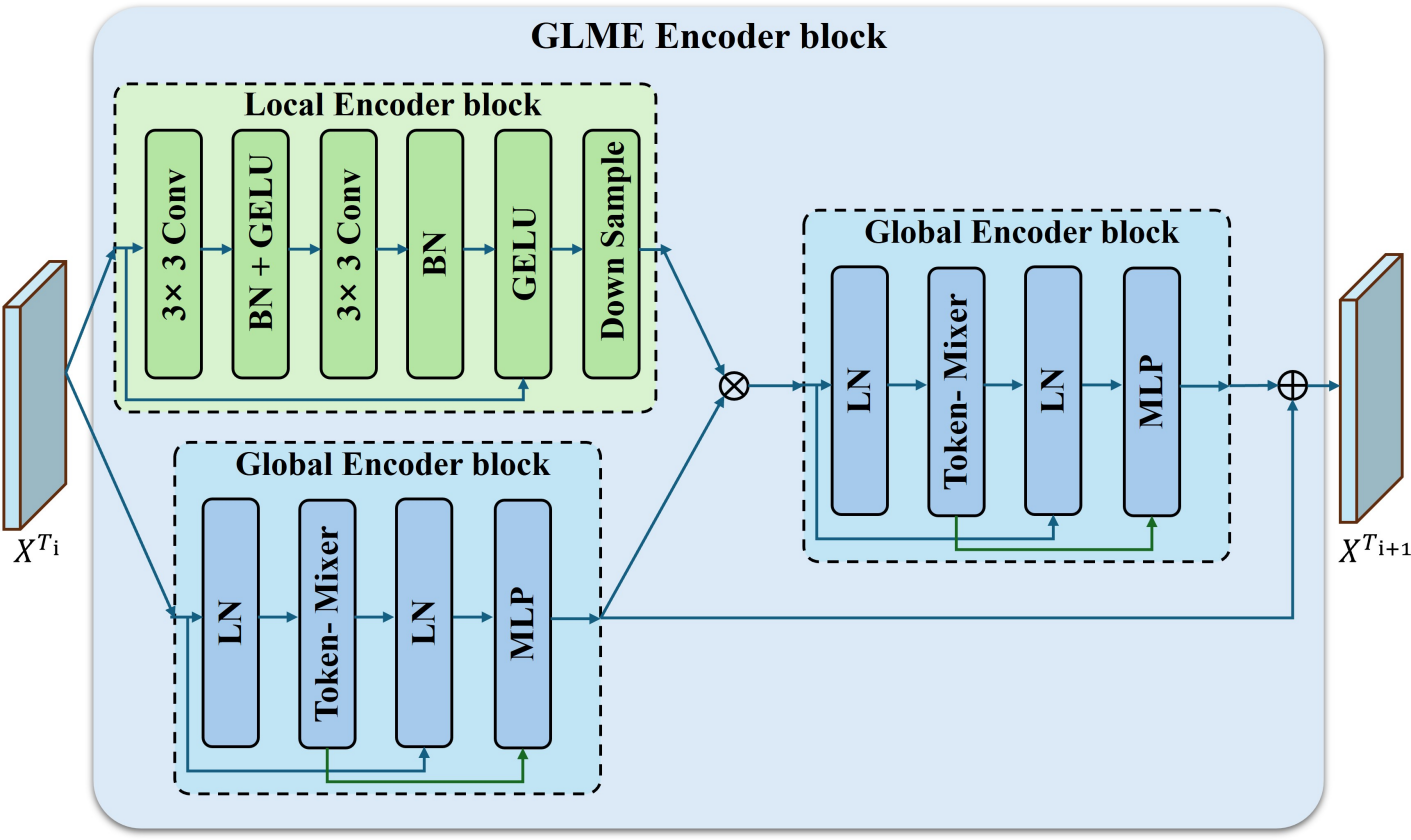
Schematic structure of the GLME module. By enhancing global and local features along separate paths and subsequently performing feature mixing, the module enables more effective extraction of discriminative representations from the input images.

To effectively integrate global contextual dependencies and local spatial details, a Global–Local Feature Mixing Embedding (GLME) module is inserted after each encoder stage. As illustrated in Figure 3, the GLME module contains parallel global and local branches, enabling long-range dependency modeling and local structure enhancement simultaneously. In this way, the module improves the discriminability of bi-temporal features and provides more informative representations for subsequent change feature fusion.

The GLME module consists of two parallel branches: a local branch (green box) and a global branch (blue box). Given an input feature map $X\in R^{H\times W\times C}$, the local branch employs stacked 3×3 convolutions, batch normalization (BN), and GELU activation to enhance local spatial details and salient responses. A downsampling operation is then applied to enlarge the receptive field and generate the local feature representation $U_{local}$.

(1) $$\begin{array}{cc} U_{1} & =\mathrm{GELU}\left(\mathrm{BN}({\mathrm{Conv}}_{3\times 3}\left(X\right))\right),\\ U_{2} & =\mathrm{GELU}\left(\mathrm{BN}({\mathrm{Conv}}_{3\times 3}\left(U_{1}\right))\right),\\ U_{local} & =\mathrm{Down}(U_{2}), \end{array}$$

The global branch adopts a residual token-mixing architecture to model long-range dependencies. Specifically, layer normalization (LN), a token-mixing operation, and an MLP are sequentially applied, while residual connections are introduced to preserve the original feature information and stabilize optimization. The process is formulated as follows:

(2) $$\begin{array}{cc} G_{1} & =X+TM(\mathrm{LN}(X)),\\ G_{2} & =G_{1}+\mathrm{MLP}\left(\mathrm{LN}\left(G_{1}\right)\right), \end{array}$$

where TM(·) denotes a lightweight token-mixing operator for spatial information exchange across different positions. It is used to capture long-range contextual dependencies in the feature map while maintaining the spatial resolution and channel dimension unchanged. Therefore, the global branch can enrich the feature representation with broader contextual awareness before feature fusion.

After spatial alignment, the outputs of the global and local branches are concatenated along the channel dimension and projected by a 1×1 convolution to obtain the enhanced feature representation:

(3) $$\hat{F}={\mathrm{Conv}}_{1\times 1}\left([G_{2}\;∥\;U_{\mathrm{local}}]\right),$$

where [·∥·] denotes channel-wise concatenation. Through this design, the GLME module jointly exploits local detail enhancement and global contextual modeling, thereby providing more discriminative features for subsequent multi-scale cross-temporal fusion.

For the *i*-th stage, the GLME module preserves the stage-wise feature dimension after fusion. Specifically, after local–global feature extraction and spatial alignment, the fused output ${\hat{F}}^{i}$ has the same spatial resolution and channel dimension as the corresponding encoder feature map. The detailed dimensional settings are summarized in Table 1.

### 3.3. The Multi-Scale Cross-Fusion Module

**Figure 4:**
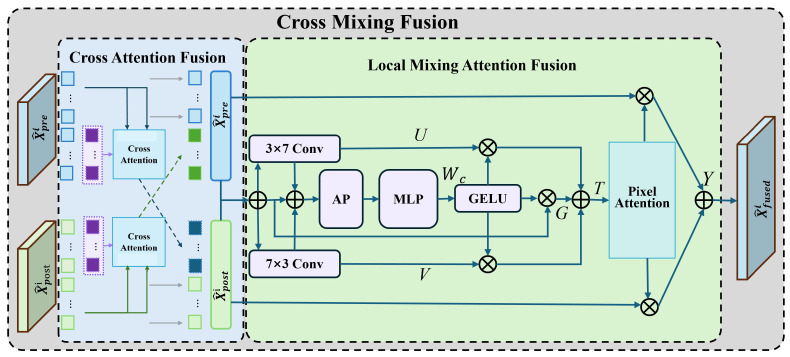
Schematic of the multi-scale cross-fusion module: the cyan section’s cross-attention and the green section’s multi-path gating and fusion functions enhance spatiotemporal positional encoding of pre- and post-change features and strengthen detection of change semantics.

As shown in Figure 4, the multi-scale cross-fusion module is designed to model deep interaction between pre-change and post-change features. Specifically, it performs cross-temporal feature interaction through a multi-head cross-attention mechanism and simultaneously aggregates multi-scale information from different encoder stages. In addition, a Local Mixing Attention (LMA) unit is introduced to preserve local structural correlations and enhance the extraction of change-related semantics.

Given the enhanced bi-temporal features, cross-attention is used to capture semantic correspondence between the two temporal branches. Its formulation is given as:

(4) $$\begin{array}{cc} \mathrm{Attention}(Q,K,V) & =\mathrm{Softmax}\left(\frac{QK^{⊤}}{\sqrt{d_{k}}}\right)V,\\ \mathrm{MHCA}(Q,K,V) & =\mathrm{Concat}\left(head_{1},\ldots ,head_{h}\right)W^{O},\\ head_{i} & =\mathrm{Attention}(QW_{i}^{Q},KW_{i}^{K},VW_{i}^{V}), \end{array}$$

Here, *Q*, *K*, and *V* are generated from the enhanced pre-change and post-change features to model cross-temporal dependency.

To capture change patterns with different spatial extents, cross-fusion is performed at each encoder stage and the resulting features are further aggregated across scales. For the *i*-th stage, the cross-fused feature can be written as

(5) $$S^{i}=\mathrm{Conv}\left(\mathrm{MHCA}({\hat{F}}_{pre}^{i},{\hat{F}}_{post}^{i},{\hat{F}}_{post}^{i})\right),$$

where $S^{i}$ denotes the intermediate cross-temporal fused feature at stage *i*.

Based on $S^{i}$, two asymmetric convolution branches are introduced to enhance directional structural modeling:

(6) $$\begin{array}{cc} U^{i} & ={\mathrm{Conv}}_{3\times 7}\left(S^{i}\right),\\ V^{i} & ={\mathrm{Conv}}_{7\times 3}\left(S^{i}\right), \end{array}$$

where $U^{i}$ and $V^{i}$ denote the direction-aware feature maps generated from the fused feature $S^{i}$.

To further enhance informative responses, channel-wise gating is generated from the fused feature branch:

(7) $$\begin{array}{cc} W_{c}^{i} & =\mathrm{Reshape}\left(\mathrm{MLP}(\mathrm{AP}\left(U^{i}\right))\right),\\ G^{i} & =\mathrm{GELU}(W_{c}^{i}), \end{array}$$

where AP(·) denotes global average pooling, $W_{c}^{i}$ denotes the channel-wise weight vector, and $G^{i}$ is the resulting gating representation.

Then, branch interaction and pixel attention are applied to produce the final stage-wise fused feature:

(8) $$\begin{array}{cc} T^{i} & =U^{i}⊙G^{i}+V^{i}⊙G^{i},\\ A_{p}^{i} & =\sigma \left(f_{pa}\left(T^{i}\right)\right),\\ Y^{i} & =T^{i}⊙A_{p}^{i},\\ F_{fused}^{i} & ={\hat{F}}_{pre}^{i}+Y^{i}+{\hat{F}}_{post}^{i}, \end{array}$$

where ⊙ denotes element-wise multiplication, σ(·) is the sigmoid function, and $f_{pa}\left(\cdot \right)$ denotes the pixel attention operation.

Unless otherwise stated, the intermediate variables in the multi-scale cross-fusion module, including $S^{i}$, $U^{i}$, $V^{i}$, $G^{i}$, $T^{i}$, $Y^{i}$, and $F_{fused}^{i}$, follow the stage-wise spatial resolution of the corresponding encoder feature map. Their dimensional relationships and the decoder configuration are summarized in Table 1.

The multi-scale cross-fusion module is designed according to a coarse-to-fine collaborative principle rather than a simple stacking of multiple attention operations. Specifically, the multi-head cross-attention (MHCA) is first introduced to explicitly model long-range semantic correspondence between pre-change and post-change features, which is essential for suppressing pseudo changes caused by seasonal variation, illumination differences, and background disturbance. However, although MHCA is effective for cross-temporal semantic alignment, it is relatively weak in preserving local structural continuity and anisotropic boundary details.

To compensate for this limitation, the local refinement branch further introduces asymmetric convolutions, channel attention, and pixel attention in sequence. The asymmetric convolutions (3×7 and 7×3) are used to capture directional and elongated local structures more efficiently than standard square kernels, which is particularly suitable for irregular mining boundaries and narrow fragmented change regions. On this basis, channel attention adaptively reweights feature channels to emphasize change-relevant semantic responses and suppress redundant or noisy feature dimensions. Finally, pixel attention performs fine-grained spatial reweighting, enabling the network to focus on subtle changed pixels and boundary transitions. Therefore, these components are functionally complementary rather than redundant: MHCA mainly addresses cross-temporal semantic interaction, asymmetric convolutions enhance local directional structure modeling, channel attention performs semantic selection in the channel dimension, and pixel attention refines spatial localization at the pixel level.

### 3.4. Loss Function

The change detection task is formulated as a pixel-wise binary classification problem, where each pixel is labeled as changed or unchanged. Let $p_{i}$ denote the predicted probability of the *i*-th pixel belonging to the changed class, and let $y_{i}\in \left\{0,1\right\}$ denote the corresponding ground-truth label. The prediction is obtained from the decoder output through a classification layer followed by a softmax operation.

(9) $$p_{i}=\mathrm{Softmax}\left(\mathrm{Cls}(F_{d,i})\right),$$

where $F_{d,i}$ denotes the decoder feature at pixel *i*, and Cls(·) denotes the final 1×1 convolutional classification layer.

In Equation (9), $F_{d,i}$ denotes the decoder feature corresponding to pixel *i*, and $p_{i}$ represents the predicted probability distribution over the changed and unchanged classes.

The overall loss function is defined as the pixel-wise binary cross-entropy loss:

(10) $$L=-\frac{1}{N}\sum_{i=1}^{N}\left[y_{i}\log\left(p_{i}\right)+\left(1-y_{i}\right)\log\left(1-p_{i}\right)\right],$$

In Equation (10), *N* is the total number of pixels. The network parameters are optimized by minimizing this loss so as to improve the accuracy of pixel-wise change prediction.

## 4. Experiment Results

### 4.1. Datasets

Model performance was evaluated on two public benchmark datasets for mining area change detection.

(1) **MACD** [40]: This is the first dataset specifically constructed for mining area change detection. It covers diverse scale variations and complex change patterns, with a focus on land-use and land-cover changes caused by mining activities, such as deforestation, erosion, and industrial expansion. The average temporal interval between the bi-temporal images exceeds three years, thus providing sufficient temporal variation for change analysis.
(2) **MineNetCD** [20]: This is a global high-resolution open-pit mining change detection dataset containing 100 representative active mining sites. The bi-temporal images have a spatial resolution of 1.2 m and were collected via Google Earth Engine. The dataset contains more than 70,000 paired image patches and covers a total area of approximately 6756.88 km^2^.

To ensure a fair comparison, we follow the official or commonly adopted data partition protocols of the two datasets. For MACD, the original split with 1801 training pairs and 332 testing pairs is directly adopted. Since MACD does not provide a standard validation set, no additional validation subset is constructed from the test set. Hyper-parameter settings are fixed across experiments to avoid biasing the evaluation.

For MineNetCD, the dataset is split at the mining site level into 60% training sites, 10% validation sites, and 30% testing sites, corresponding to 47,743, 4613, and 19,355 cropped patch pairs, respectively. The validation set is used for model selection and hyper-parameter tuning, while the test set is used only for final performance evaluation.

### 4.2. Evaluation Indicators

To evaluate the performance of different methods, four metrics are adopted in this study, including Precision, F1-score, Intersection over Union (IoU), and Overall Accuracy (OA). For all these metrics, higher values indicate better change detection performance. Their definitions are given as follows:

(11) $$\begin{array}{cc} \mathrm{Precision} & =\frac{\mathrm{TP}}{\mathrm{TP}+\mathrm{FP}},\\ \mathrm{Recall} & =\frac{\mathrm{TP}}{\mathrm{TP}+\mathrm{FN}},\\ F_{1} & =\frac{2\times \mathrm{Precision}\times \mathrm{Recall}}{\mathrm{Precision}+\mathrm{Recall}},\\ \mathrm{IoU} & =\frac{\mathrm{TP}}{\mathrm{TP}+\mathrm{FP}+\mathrm{FN}},\\ \mathrm{OA} & =\frac{\mathrm{TP}+\mathrm{TN}}{\mathrm{TP}+\mathrm{FP}+\mathrm{TN}+\mathrm{FN}},\\ P_{e} & =\frac{\left(\mathrm{TP}+\mathrm{FP})(\mathrm{TP}+\mathrm{FN})+(\mathrm{FN}+\mathrm{TN})(\mathrm{FP}+\mathrm{TN}\right)}{{\left(\mathrm{TP}+\mathrm{FP}+\mathrm{TN}+\mathrm{FN}\right)}^{2}} \end{array}$$

where TP (true positives) denotes the number of changed pixels correctly detected as change, TN (true negatives) denotes the number of unchanged pixels correctly detected as no-change, FP (false positives) denotes the number of unchanged pixels incorrectly classified as change, and FN (false negatives) denotes the number of changed pixels incorrectly classified as no-change.

### 4.3. Implementation Details

The encoder produces feature maps with channel dimensions of 64, 96, 128, and 256, respectively, given co-registered bi-temporal inputs of size 256×256×3. The decoder outputs a binary change map with the same spatial resolution as the input.

During training, several data augmentation strategies are applied, including random flipping, scale-aware cropping, color jittering, and Gaussian blurring. The network is trained for 300 epochs on four NVIDIA RTX 3090 GPUs using the Adam optimizer with a weight decay of 0.01. The initial learning rate is set to $3.1\times 10^{-4}$ and linearly decayed to zero throughout training.

In addition, the shared-weight Siamese encoder reduces model redundancy and is beneficial for improving generalization across bi-temporal branches. Normalization layers (BN/LN) and residual connections are also adopted to stabilize optimization and enhance training robustness. No additional explicit regularization techniques, such as dropout or stochastic depth, are used unless otherwise specified.

### 4.4. Experimental Results and Analysis

Table 2 shows that GLMECD-Net outperforms all compared state-of-the-art (SOTA) methods on the MineNetCD dataset across all evaluation metrics. In particular, the IoU reaches 53.82%, improving upon ELGC-Net (52.4%), TransUNetCD (48.57%) [41], and MineNetCD (50.19%) by 1.40%, 5.25%, and 3.63% [20], respectively. The F1-score is 77.53%, which is substantially higher than those of the competing approaches.

**Figure 5:**
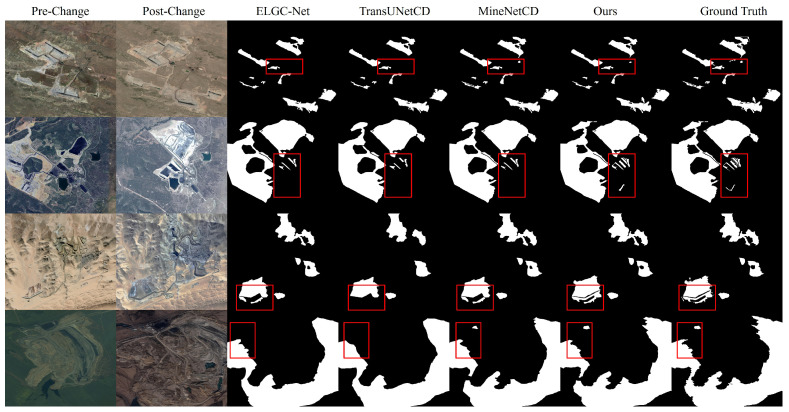
Qualitative comparison on the MineNetCD dataset with three recent state-of-the-art change detection methods. The red boxes highlight representative challenging local regions, including thin and fragmented change structures, small change targets, and boundary details under seasonal appearance variations. These highlighted areas clearly illustrate the differences among methods in fine-grained detail preservation, boundary delineation, and robustness to cross-season image discrepancies.

As illustrated by the qualitative comparisons with the other three SOTA change detection methods in Figure 5, GLMECD-Net can accurately detect subtle changes that ELGC-Net and TransUNetCD fail to capture (e.g., scattered reclamation parcels and small relocated spoil heaps). It demonstrates superior performance in identifying changes within small-area regions, whereas the competing methods exhibit pronounced omission errors. In particular, the red boxes mark several challenging local regions containing thin change traces, fragmented small targets, and irregular boundary details. In these highlighted areas, the competing methods often exhibit missed detections, incomplete extraction, or blurred boundaries, whereas our method preserves more complete local structures and produces predictions that are closer to the ground truth, demonstrating stronger fine-grained detection capability and better robustness under seasonal variations.

Table 3 shows that GLMECD-Net achieves the best overall performance on the MACD dataset among the compared methods. In particular, it obtains the highest IoU of 53.82%, outperforming ELGC-Net (52.43%), TransUNetCD (48.57%), and MineNetCD (50.19%) by 1.39%, 5.25%, and 3.63%, respectively. It also achieves the highest F1-score of 77.53% and the highest OA of 83.78%. Although its Precision is slightly lower than that of ELGC-Net, the overall results indicate a better balance between false positives and false negatives.

**Figure 6:**
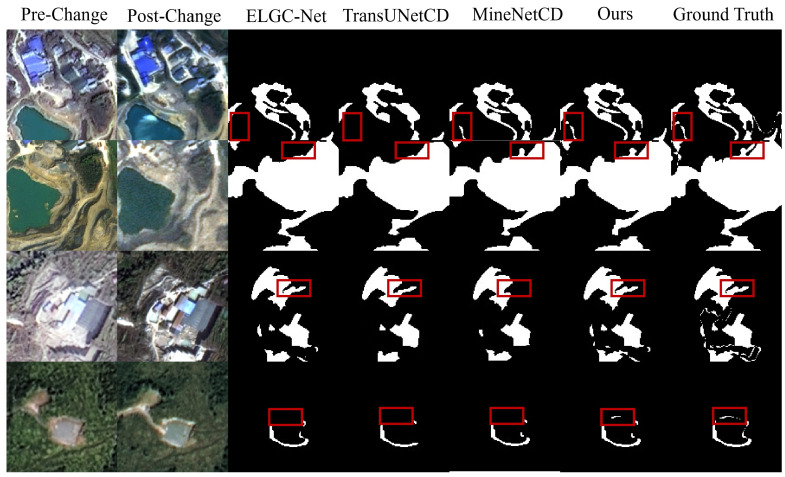
Qualitative comparison on the MACD dataset with three recent state-of-the-art change detection methods. The red boxes highlight challenging local regions involving thin structures, fragmented small changes, and irregular boundaries, where the differences in detail preservation and boundary delineation can be clearly observed.

As shown in Figure 6, when compared with the other three SOTA change detection methods, GLMECD-Net can effectively identify true change regions while suppressing false alarms, and it exhibits particularly strong performance in capturing subtle change details. In contrast, the competing approaches (ELGC-Net, TransUNetCD, and MineNetCD) produce a considerable number of false positives and false negatives, making it difficult for them to accurately delineate the boundaries of fragmented change regions. In particular, the red boxes highlight several challenging regions containing thin change traces, small fragmented targets, and irregular boundary structures. In these areas, ELGC-Net, TransUNetCD, and MineNetCD tend to miss subtle changed pixels, generate incomplete responses, or produce over-smoothed boundaries, whereas our method preserves finer structural details and yields more accurate boundary delineation. These results demonstrate that the proposed network has stronger capability in capturing subtle and heterogeneous change patterns in complex mining scenes.

Table 4 reports the numbers of parameters and FLOPs of different methods for efficiency comparison. Our method uses 50 M parameters and 8.72 G FLOPs, which shows that although the model size is not the smallest, its computational cost is lower than that of the compared methods. This suggests that the proposed architecture improves change detection performance while maintaining competitive computational efficiency.

### 4.5. Ablation Study

As shown in Table 5, the effectiveness of the local-enhanced encoder and multi-channel aggregation in the GLME module is evaluated on the MACD dataset. When only the multi-channel aggregation component is enabled, the IoU reaches 54.24%. When only the local-enhanced encoder is enabled, the IoU is 53.32%. In contrast, the complete GLME module that integrates both components achieves the best IoU of 56.67%, demonstrating that the joint modeling of complementary feature representations is important for improving overall detection performance.

The effectiveness of the cross-attention mechanism and the local attention component is evaluated in Table 6. Enabling the cross-attention mechanism increases the IoU from 52.89% to 54.62%, indicating that explicit cross-temporal semantic interaction is beneficial for establishing correspondence between bi-temporal features. When the local attention component is used alone, the IoU reaches 55.11%, showing that local structural refinement is also important for capturing subtle boundary details and fragmented change patterns. Furthermore, the complete multi-scale cross-fusion module that integrates both components achieves the best IoU of 56.67%, which is 3.78% higher than the baseline. This result suggests that the two components are complementary rather than redundant: cross-attention mainly improves semantic correspondence across time, while the local refinement branch mainly enhances intra-feature structural discrimination and boundary localization.

## 5. Discussion

### 5.1. Model Performance Advantages and Core Mechanisms

The superior performance of GLMECD-Net on mining area change detection tasks primarily arises from the synergistic effect of three core designs.

First, the multi-stage Siamese encoder extracts hierarchical features at different spatial scales, which helps the network adapt to the pronounced scale heterogeneity in mining scenarios, where large open-pit regions and scattered reclamation parcels often coexist. This multi-scale representation is beneficial for reducing missed detections in large change areas while preserving sensitivity to small and fragmented changes.

Second, the GLME module aggregates global contextual information and local spatial details in parallel. This design enlarges the effective receptive field while preserving fine-grained structural cues, which is particularly important for mining area change detection, where both large-area disturbances and subtle boundary variations are common. The ablation results further indicate that combining the two branches yields better performance than using either component alone, highlighting the complementary roles of global dependency modeling and local detail enhancement.

Finally, the multi-scale cross-fusion module strengthens cross-temporal feature interaction through cross-attention, thereby improving the discriminability of change-related semantics. By explicitly modeling the correspondence between pre-change and post-change features, this module helps suppress interference caused by illumination variation, seasonal differences, and other nuisance factors. In addition, the local attention component further enhances structural consistency and improves the representation of subtle change patterns.

### 5.2. Essential Differences from Existing Methods

Compared with existing CNN-based and Transformer-based approaches, the core advantage of GLMECD-Net lies in overcoming the traditional dichotomy between “local feature extraction” and “global context modeling”:

(1) Conventional CNN-based methods (e.g., ELGC-Net) are constrained by limited receptive fields and thus struggle to capture long-range dependencies associated with large-scale changes in mining areas, leading to blurred delineation along the boundaries of large open-pit regions. In contrast, GLMECD-Net leverages the global branch in the GLME module to enable effective interaction among distant features, yielding a 3.67% improvement in IoU over large-area change regions.
(2) Existing Transformer-based methods (e.g., TransUNetCD) are effective at modeling global context but may be less sensitive to fine local details, especially in small and fragmented change regions. GLMECD-Net addresses this issue by incorporating a local enhancement branch, which strengthens fine-grained feature extraction while preserving the global modeling ability of the overall architecture.
(3) Most existing approaches perform feature fusion via simple concatenation or element-wise addition, whereas the multi-scale cross-fusion module in GLMECD-Net adopts attention mechanisms to adaptively reweight features, enabling deep interactions across time and scale. This design substantially improves the model’s robustness to complex disturbances such as shadows and variations in vegetation cover.

### 5.3. Application Scenarios and Practical Value

The high-precision detection capability of GLMECD-Net endows it with broad application prospects in mining area ecological monitoring and management:

(1) In dynamic monitoring of mining activities, the model can accurately identify changes such as mining area expansion and spoil heap relocation, providing technical support for mineral resource development planning and compliance supervision, and effectively constraining illegal mining activities.
(2) In ecological and environmental assessment, GLMECD-Net enables quantitative monitoring of changes related to vegetation restoration and soil erosion, thereby providing data support for the formulation and performance evaluation of ecological restoration schemes, and facilitating the protection and rehabilitation of mining area ecosystems.
(3) In land-use planning, the model can rapidly extract information on land-use-type changes within mining areas, offering decision-making references for optimal allocation of land resources and sustainable land management.

### 5.4. Limitations and Future Directions

Despite its superior performance, GLMECD-Net still has several limitations:

(1) The computational complexity of the model remains relatively high, making it difficult to meet real-time detection requirements on edge devices, and its efficiency in large-scale mining area monitoring needs further improvement.
(2) The model is sensitive to image quality degradation caused by extreme weather conditions (e.g., heavy rainfall, sandstorms), which can adversely affect detection performance.
(3) The training process relies heavily on annotated data, whereas acquiring high-quality change labels in mining areas is costly and time-consuming.

Future research will focus on the following directions:

(1) Introducing lightweight network designs (e.g., MobileNet, ShuffleNet backbones) and model compression techniques to reduce computational complexity and promote deployment on edge devices.
(2) Designing adaptive image quality enhancement modules to improve robustness under adverse weather conditions.
(3) Exploring semi-supervised and weakly supervised learning strategies to reduce dependence on labeled data and lower model training costs.
(4) Extending to multi-modal data fusion (e.g., optical remote sensing, SAR imagery, and LiDAR data) to further enhance change detection accuracy in complex scenarios.

## 6. Conclusions

This paper addresses the complex requirements of change detection in open-pit mining areas using remote sensing imagery and proposes an efficient detection model, GLMECD-Net, based on global–local feature fusion. Through the synergistic design of a multi-scale, multi-stage Siamese encoder, the GLME module, and a multi-scale cross-fusion module, the model achieves deep integration of global contextual information and local fine-grained features, thereby significantly improving the detection accuracy for multi-scale, irregular, and fragmented changes in mining areas. Experiments on two public datasets, MACD and MineNetCD, demonstrate that GLMECD-Net attains state-of-the-art performance in terms of core metrics including Precision, Recall, F1-score, and IoU, and is capable of accurately capturing both subtle and large-scale changes in mining regions. This provides an innovative technical solution for mining activity monitoring, ecological environmental protection, and sustainable land management. With further research on model lightweighting, robustness enhancement, and multi-modal data fusion, GLMECD-Net is expected to enable real-time, accurate, and low-cost monitoring of mining area changes, offering stronger technical support for ecological civilization construction in mining regions.

## Figures and Tables

**Table 1:** Detailed configuration of GLMECD-Net for reproducibility. Here, the input image size is 256×256×3, and the same encoder configuration is used for both temporal branches.

Module	Stage	Operation/Parameter	Input Size	Output Size
Input	–	Co-registered bi-temporal images	2×256×256×3	2×256×256×3
Encoder	Stage 1	Patch embedding + encoder block + GLME	256×256×3	64×64×64
Encoder	Stage 2	Patch embedding + encoder block + GLME	64×64×64	32×32×96
Encoder	Stage 3	Patch embedding + encoder block + GLME	32×32×96	16×16×128
Encoder	Stage 4	Patch embedding + encoder block + GLME	16×16×128	8×8×256
GLME local branch	each stage *i*	3×3 Conv + BN + GELU, repeated twice; Down(·)	$H_{i}\times W_{i}\times C_{i}$	aligned to $H_{i}\times W_{i}\times C_{i}$
GLME global branch	each stage *i*	LN → TM → residual → LN → MLP → residual	$H_{i}\times W_{i}\times C_{i}$	$H_{i}\times W_{i}\times C_{i}$
GLME fusion	each stage *i*	Concatenation + 1×1 Conv	$H_{i}\times W_{i}\times 2C_{i}$	$H_{i}\times W_{i}\times C_{i}$
Cross-fusion	each stage *i*	MHCA + Conv	$H_{i}\times W_{i}\times C_{i}$	$H_{i}\times W_{i}\times C_{i}$
LMA/directional branch	each stage *i*	3×7 Conv and 7×3 Conv + AP + MLP + pixel attention	$H_{i}\times W_{i}\times C_{i}$	$H_{i}\times W_{i}\times C_{i}$
Stage-wise fusion output	each stage *i*	Residual fusion of ${\hat{F}}_{pre}^{i}$, $Y^{i}$, and ${\hat{F}}_{post}^{i}$	$H_{i}\times W_{i}\times C_{i}$	$H_{i}\times W_{i}\times C_{i}$
Prediction head	–	Final convolution layer + Argmax	$256\times 256\times C_{d}$	256×256×2→256×256

**Table 2:** Comparison results on the MineNetCD dataset with three recent state-of-the-art change detection algorithms. The best data is highlighted in bold in the table.

Method	F1 ↑	IoU ↑	Precision ↑	OA ↑
ELGC-Net	73.18	52.43	**72.04**	83.45
TransUnetCD	54.92	48.57	62.25	80.01
MineNetCD	74.58	50.19	69.94	82.56
GLMECD-Net	**77.53**	**53.82**	71.66	**83.78**

**Table 3:** Comparison results on the MACD dataset with three recent state-of-the-art change detection algorithms.The best data is highlighted in bold in the table.

Method	F1 ↑	IoU ↑	Pre ↑	OA ↑
ELGC-Net	67.74	53.00	70.14	82.84
TransUnetCD	61.43	45.44	68.57	79.99
MineNetCD	62.26	52.92	74.73	83.73
GLMECD-Net	**70.25**	**56.67**	**75.86**	**84.11**

**Table 4:** Quantitative comparison of model complexity, including parameters and FLOPs, on the MineNetCD dataset. The best data is highlighted in bold in the table.

Method	Parameters (M) ↓	Flops (G) ↓
ELGC-Net [19]	**10.57**	12.35
MCAT [40]	10.64	34.94
MineNetCD [20]	89	15.46
GLMECD-Net	50	**8.72**

**Table 5:** Ablation study metrics of the GLME module on the MACD dataset.

Local-Enhanced Encoder	Multi-Channel Aggregation	F1 ↑	IoU ↑	OA ↑
		62.71	49.33	75.50
	🗸	66.18	54.24	81.03
🗸		68.10	53.32	80.08
🗸	🗸	70.25	56.67	84.11

**Table 6:** Ablation study metrics of the cross-attention mechanism and local hybrid attention in the cross-attention module on the MACD dataset.

Cross-Attention Model	Local Hybrid Attention	F1	IoU	OA
		66.01	52.89	76.58
	🗸	67.99	55.11	77.23
🗸		68.70	54.62	78.49
🗸	🗸	70.25	56.67	84.11

## Data Availability

The data that support the findings of this study are openly available at https://github.com/chh11/MCAT (accessed on 10 October 2021) and https://github.com/EricYu97/MineNetCD (accessed on 4 July 2024).
